# Hormonal crosstalk for root development: a combined experimental and modeling perspective

**DOI:** 10.3389/fpls.2014.00116

**Published:** 2014-03-27

**Authors:** Junli Liu, James Rowe, Keith Lindsey

**Affiliations:** The Integrative Cell Biology Laboratory, School of Biological and Biomedical Sciences, The Biophysical Sciences Institute, Durham UniversityDurham, UK

**Keywords:** root development, POLARIS peptide, hormonal crosstalk, osmotic stress, kinetic modeling

## Abstract

Plants are sessile organisms and therefore they must adapt their growth and architecture to a changing environment. Understanding how hormones and genes interact to coordinate plant growth in a changing environment is a major challenge in developmental biology. Although a localized auxin concentration maximum in the root tip is important for root development, auxin concentration cannot change independently of multiple interacting hormones and genes. In this review, we discuss the experimental evidence showing that the **POLARIS peptide** of Arabidopsis plays an important role in **hormonal crosstalk** and root growth, and review the crosstalk between auxin and other hormones for root growth with and without osmotic stress. Moreover, we discuss that experimental evidence showing that, in root development, hormones and the associated regulatory and target genes form a network, in which relevant genes regulate hormone activities and hormones regulate gene expression. We further discuss how it is increasingly evident that mathematical modeling is a valuable tool for studying **hormonal crosstalk**. Therefore, a combined experimental and modeling study on **hormonal crosstalk** is important for elucidating the complexity of root development.

## Introduction

Hormone signaling systems coordinate plant growth and development through a range of complex interactions. The activities of hormones such as auxin, ethylene, cytokinin, abscisic acid, gibberellin, and brassinosteroids depend on cellular context and exhibit either synergistic or antagonistic interactions. Additionally, auxin is directionally transported through plant tissues, providing positional and vectorial information during development (Vanneste and Friml, 2009). Patterning in Arabidopsis root development is coordinated via a localized auxin concentration maximum in the root tip (Sabatini et al., 1999), requiring the regulated expression of specific genes. This auxin gradient has been hypothesized to be sink-driven (Friml et al., 2002) and computational modeling suggests that auxin efflux carrier activity may be sufficient to generate the gradient in the absence of auxin biosynthesis in the root (Grieneisen et al., 2007; Wabnik et al., 2010). However, other experimental studies show that local auxin biosynthesis modulates gradient-directed planar polarity in Arabidopsis, and a local source of auxin biosynthesis contributes to auxin gradient homeostasis (Ikeda et al., 2009). Thus genetic studies show that auxin biosynthesis (Ikeda et al., 2009; Normanly, 2010; Zhao, 2010), the AUX1/LAX influx carriers (Swarup et al., 2005, 2008; Jones et al., 2008; Krupinski and Jonsson, 2010), and the PIN auxin efflux carriers (Petrásek et al., 2006; Grieneisen et al., 2007; Krupinski and Jonsson, 2010; Mironova et al., 2010) all play important roles in the formation of auxin gradients.

In addition, experimental evidence shows that, in root development, hormones and the associated regulatory and target genes form a network, in which relevant genes regulate hormone activities and hormones regulate gene expression. For example, ethylene promotes auxin flux in the root, in a process dependent on the **POLARIS (PLS) peptide** (Ruzicka et al., 2007; Swarup et al., 2007; Liu et al., 2010a). Furthermore, PIN levels are positively regulated by ethylene and auxin in Arabidopsis roots (Ruzicka et al., 2007). Interestingly, cytokinin can negatively regulate PIN levels (Ruzicka et al., 2009), while repressing auxin biosynthesis and promoting ethylene responses (Nordstrom et al., 2004; Chandler, 2009; Liu et al., 2010a). Cytokinin also has the capacity to modulate auxin transport, by transcriptional regulation of the *PIN* genes (Ruzicka et al., 2009).

KEY CONCEPT 1. POLARIS (PLS) peptide The POLARIS (PLS) peptide was identified in the plant species *Arabidopsis thaliana* by promoter trapping. The gene encodes a mRNA of ca. 600 bases, at the 3'-end of which is a 36 amino acid open reading frame (ORF). Translation of the ORF is required for biological activity. The transcriptional start of the gene overlaps with the 3'-UTR of an upstream gene. Expression of the *PLS* gene is strongest in root tips, but is also detectable in young leaves, and is induced by auxin. Mutation of *POLARIS* leads to several developmental defects, including a short root phenotype and reduced vascular complexity in leaves. Recent results show a role for PLS in repressing ethylene responses, and in promoting ethylene-mediated auxin biosynthesis.

Different aspects of **hormonal crosstalk** in root development have been reviewed recently (Bishopp et al., 2011; Depuydt and Hardtke, 2011; Ross et al., 2011; Garay-Arroyo et al., 2012; Hwang et al., 2012; Vanstraelen and Eva Benkova, 2012). These review articles have concentrated on the interactions of either a wide range of hormones or the specific interactions of a couple of hormones. For example, the review by Garay-Arroyo et al. (2012) covers auxin, ethylene, cytokinin, gibberellins, brassinosteroids and abscisic acid. The review by Hwang et al. (2012) mainly discusses the interaction between cytokinin and auxin in detail. The readers may consult those reviews for different aspects of **hormonal crosstalk**. Here our focused review concentrates on a combined experimental and modeling perspective of **hormonal crosstalk** in root development.

KEY CONCEPT 2. Hormonal crosstalk Hormonal crosstalk refers to the phenomenon whereby the activities of hormones such as auxin, ethylene, cytokinin, abscisic acid, gibberellin, and brassinosteroids exhibit either synergistic or antagonistic interactions, depending on cellular context.

## Roles of the polaris peptide of arabidopsis in hormonal crosstalk and root growth

**Figure 1:**
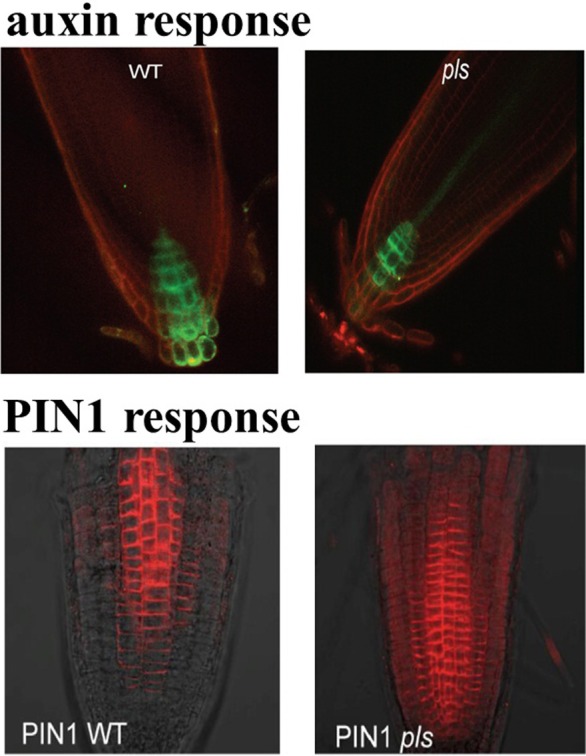
**DR5::GFP expression in wild type and *pls* mutant, showing difference in auxin gradients (upper panel), and PIN1 immunolocalization in wildtype and *pls* mutant, showing differences in PIN protein levels (lower panel)**. This figure is adapted with permission from the Figure 4 of Liu et al. (2010a) and the Figure 1 of Liu et al. (2013).

The *POLARIS* (*PLS*) gene of Arabidopsis transcribes a short mRNA encoding a 36-amino acid peptide (Casson et al., 2002). Expression of the *PLS* gene of *Arabidopsis* is repressed by ethylene and induced by auxin (Casson et al., 2002; Chilley et al., 2006). It was also experimentally shown that *pls* mutant roots are short, with reduced cell elongation, and they are hyper-responsive to exogenous cytokinins. Moreover, *pls* mutant roots show increased expression of the cytokinin-inducible gene, *ARR5/IBC6*, compared with the wild type (Casson et al., 2002). On the other hand, in the *pls* mutant, auxin concentration is reduced (Figure 1), cytokinin concentration is enhanced and ethylene production remains approximately unchanged compared to wild-type (Casson et al., 2002; Chilley et al., 2006; Liu et al., 2010a). In the *PLS* overexpressing transgenic *PLOSox*, auxin concentration is increased, while ethylene production remains approximately unchanged. In the ethylene resistant *pls etr1* double mutant, auxin concentration is approximately recovered to the same level as that in wild-type seedlings (Casson et al., 2002; Chilley et al., 2006; Liu et al., 2010a). In addition, immunolocalization studies reveal that both PIN1 (Figure 1) and PIN2 protein levels increase in the *pls* mutant, and decrease in *PLSox* (Liu et al., 2013). In the ethylene-insensitive *etr1* mutant, PIN1 and PIN2 levels are lower than those in wild-type. In addition, the double mutant *pls etr1* exhibits reduced PIN1 and PIN2 levels compared to *pls* and slightly lower PIN1 and PIN2 levels compared to wild-type (Liu et al., 2013). Therefore, experimental data have shown that the *PLS* gene plays important roles in the crosstalk between auxin, ethylene and cytokinin.

**Figure 2:**
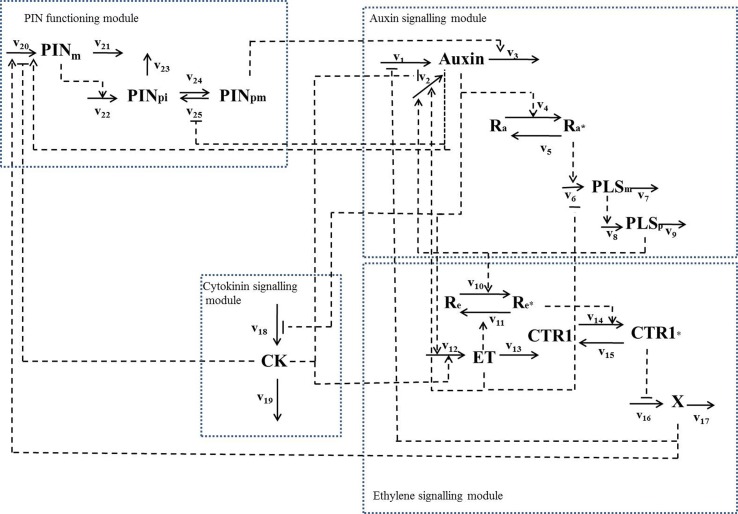
**A hormonal crosstalk network of auxin, ethylene and cytokinin for root development, showing that change in one signaling component leads to change in other signaling components in the network (modified with permission from Liu et al., 2013)**. The reaction rates are: v1, total auxin influx from all neighboring; v2, auxin biosynthesis rate in the cell; v3, total auxin efflux from the cell; v4, rate for conversion of the inactive form of the auxin receptor, Ra, to its active form, Ra^*^; v5, rate for conversion of the active form of the auxin receptor, Ra^*^, to its inactive form, Ra; v6, transcription rate of the POLARIS (PLS)gene; v7, decay rate of PLS mRNA; v8, translation rate of the PLS protein; v9, decay rate of PLS protein; v10, rate for conversion of the inactive form of the ethylene receptor, Re, to its active form by PLS protein (PLSp), Re^*^; v11, rate for conversion of the active form of ethylene receptor, Re^*^, to its inactive form, Re; v12, ethylene biosynthesis rate; v13, rate for removal of ethylene; v14, rate for conversion of the inactive form of the CONSTITUTIVE TRIPLE RESPONSE 1 (CTR1) protein, CTR1, to its active form, CTR1^*^; v15, rate for conversion of the active form of CTR1 protein, CTR1^*^, to its inactive form, CTR1; v16, rate for activation of the ethylene signaling response; v17, rate for removal of the unknown ethylene signaling component, X; v18, rate for cytokinin biosynthesis; v19, rate for removal of cytokinin; v20, transcription rate of the PIN gene; v21, rare for the decay of PIN mRNA; v22, translation rate of PIN protein; v23, rate for decay of PIN protein in cytosol; v24, rate for transport of PIN protein from cytosol to plasma membrane; v25, rate for internalization of PIN protein. When exogenous hormones are applied: v26, rate for uptake of IAA when exogenous IAA is applied; v27, rate for uptake of ACC when exogenous ACC is applied; v28, rate for uptake of cytokinin when exogenous cytokinin is applied.

By combining the experimental data relating to the *PLS* gene with a variety of other experimental data in the literature, we have revealed that PLS, PIN1/PIN2, and three hormones (auxin, ethylene and cytokinin) form an interacting network (Figure 2), in which expression of *PLS* and PIN1/PIN2 levels regulate auxin, ethylene and cytokinin responses, which in turn regulate expression of *PLS* and PIN1/PIN2 (Liu et al., 2013). In addition, changing the concentration of, or response to a given hormone may also change the concentrations of/responses to other hormones. Therefore, functions of hormones and the associated genes in root development must be analyzed as an integrative system, as exemplified in Figure 2.

## Crosstalk between auxin and other hormones for root growth

Figure 2 describes the crosstalk network for three major hormones: auxin, cytokinin and ethylene. This network can be further expanded by including other crosstalk components. At early developmental stages, the balance between auxin and cytokinin signaling is crucial: for example, in the Arabidopsis root meristem, auxin promotes cell division (Dello Ioio et al., 2008) and cytokinin promotes cell differentiation (Perilli et al., 2010). Moubayidin et al. (2010) found that higher gibberellin (GA) levels in the young meristem repress *ARR1* expression, which is accompanied by a drop in *IAA3/SHY2* transcription. The ARR1 cytokinin-responsive transcription factor activates the gene *SHY2*, which negatively regulates the *PIN* genes encoding auxin transport facilitators. Thus, GA forms a circuit regulating the balance between auxin and cytokinin signaling.

Brassinosteroids (BRs) also interact with auxin. For example, both BRs and auxin pathways synergistically regulate the expression of several auxin-responsive genes (Mouchel et al., 2006). BRs also regulate root development and this activity is concentration-dependent (Mussig et al., 2003). Thus, a complex relationship between auxin and BRs is formed (Hardtke, 2007).

Abscisic acid (ABA) is an isoprenoid hormone that is involved in the regulation of seed development and dormancy, as well as plant responses to various environmental stresses (Finkelstein and Rock, 2002). It has been experimentally shown that the gene *VIVIPAROUS1 (VP1)* in maize and its Arabidopsis ortholog *ABI3*, which encodes a transcription factor involved in ABA signaling, is auxin-inducible (Suzuki et al., 2001; Brady et al., 2003). Therefore, ABA interacts with the crosstalk network via the action of auxin (Figure 2).

The interaction between auxin, cytokinin and ethylene in Figure 2 may also be extended to include other signals that regulate root development. For example, glucose signals regulate Arabidopsis seedling root directional growth by interacting with auxin, cytokinin and ethylene (Kushwah et al., 2011). A specific hexokinase in Arabidopsis (*HXK1*) has a predominant role in glucose signaling. It has been experimentally shown that catalytically inactive *HXK1* restores auxin sensitivity for callus and root induction, indicating that the action of the HKX-dependent pathway is closely associated with the action of the auxin signaling pathway (Moore et al., 2003). Therefore, glucose signals can be integrated into the **hormonal crosstalk network** via interplay with auxin signaling.

KEY CONCEPT 3. Hormonal crosstalk network A hormonal crosstalk network is a type of biological network that describes gene expression, signal transduction and metabolic conversion complexities associated with hormonal crosstalk activity in plant development. A hormonal crosstalk network is therefore a mixed-type network that integrates transcriptomic, proteomic and metabolic networks.

## Hormonal crosstalk under osmotic stress

Plants remodel their root architecture to deal with osmotic stress, inhibiting lateral root initiation and altering root growth rates (van der Weele et al., 2000; Deak and Malamy, 2005). At low to moderate levels of osmotic stress root growth is increased and at higher levels it is inhibited (van der Weele et al., 2000; Xu et al., 2013).

Hormone crosstalk integrates stress responses with developmental control and as with most abiotic stresses, osmotic stress is characterized by an increase in abscisic acid levels. Increases in abscisic acid are concentrated in the root cap and are less significant than in aerial tissues, but are essential to normal growth under osmotic stress (Christmann et al., 2005; Deak and Malamy, 2005; Xu et al., 2013).

The increase in root growth rate under moderate osmotic stress occurs in an ABA-dependent manner (van der Weele et al., 2000; Xu et al., 2013). Osmotically induced ABA increases basipetal auxin transport through elevated PIN2 and AUX1 levels, increasing H^+^-ATPase activity and root elongation (Xu et al., 2013). High levels of applied ABA also induce expression of *ARF2*, a negative regulator of auxin responses and *arf2* mutants display altered auxin transport and shorter roots under ABA application (Wang et al., 2011).

Cytokinin is also thought to play a role in osmotic stress responses. Mature plants alter expression of cytokinin biosynthesis and metabolism genes, decreasing cytokinin levels under dehydration stress (Nishiyama et al., 2011). The AHK2 and AHK3 cytokinin receptor kinases negatively regulate ABA and osmotic stress responsive gene expression, and cytokinin-deficient mutants have increased survival rates under stress (Tran et al., 2007; Werner et al., 2010; Nishiyama et al., 2011). Intriguingly, cytokinin-deficient mutants display lower levels of ABA, implying that a positive feedback on ABA may exist (Nishiyama et al., 2011).

Osmotic stress can also promote ethylene biosynthesis, which can have an antagonistic role with ABA on root growth (Ichimura et al., 2000; Sharp, 2002; Liu and Zhang, 2004; Joo et al., 2008; Cheng et al., 2009). ABA inhibits ethylene biosynthesis by positively regulating *ERF11* via *HY5* to repress expression of *ACS5*, which catalyses the rate limiting enzyme in ethylene biosynthesis (Vogel et al., 1998; Li et al., 2011). Ethylene can also limit ABA biosynthesis, with ethylene-insensitive mutants hyperaccumulating ABA, associated with increased expression of ABA biosynthetic genes such as *NCED3* (Ghassemian et al., 2000; Chiwocha et al., 2005; Wang et al., 2007; Cheng et al., 2009).

Genetically or pharmacologically impairing ethylene signaling makes plants insensitive to root growth inhibition by ABA, but plants impaired in ethylene signaling have shorter roots under severe osmotic stress (Beaudoin et al., 2000; Ghassemian et al., 2000; Wang et al., 2007, 2008). Where limiting ethylene perception makes plants less sensitive to ABA-induced root shortening, pharmacologically limiting ethylene biosynthesis makes plants more sensitive (Ghassemian et al., 2000). This implies that both hormones are required for correct root growth under osmotic stress but the antagonistic regulation of each other's biosynthesis is insufficient to explain inhibition of root growth responses alone.

Mutant analysis has revealed little interaction between the two signal transduction pathways (Cheng et al., 2009). However *EIN2*, an essential component of ethylene signaling, shows reduced expression under osmotic stress but not ABA treatment, indicating ethylene signaling may be mediated by stress independently of ABA (Wang et al., 2007).

Osmotic stress clearly affects hormone levels in a series of complex interactions which cannot explain root growth adequately in isolation. Hormones and the associated regulatory and target genes in plant root form a network in which relevant genes regulate hormone activities and hormones regulate gene expression. An important question for understanding these complex interactions is: what are the mechanisms that regulate the fluxes of plant hormones and levels of the proteins encoded by the regulatory and target genes? To address this question, it is increasingly evident that mathematical modeling is becoming a valuable tool to tackle the complexity of **hormonal crosstalk**.

## Mathematical modeling as a valuable tool for studying hormonal crosstalk

A **hormonal crosstalk network** is a type of network consisting of gene expression, signal transduction and metabolic conversions. For example, in Figure 2, hormonal signals are transduced, auxin, ethylene, and cytokinin are synthesized and decayed through metabolic processes, and expression of *PIN* and *PLS* genes is realized. Therefore, a **hormonal crosstalk network** is a mixed-type network that integrates all these components. There are different mathematical tools available for analysing plant biological networks at different levels (Liu et al., 2010b). In particular, **kinetic modeling** (Rohwer, 2012) is useful to analyse quantitatively **hormonal crosstalk networks**.

KEY CONCEPT 4. Kinetic modeling Kinetic modeling is a modeling method that uses differential equations to analyse how each component (concentration, reaction rate) in a hormonal crosstalk network changes in space and time. In kinetic modeling, the rate of a reaction is described by the concentrations of all chemicals involved in the reaction, and the mass balance of all chemicals is described using differential equations.

The **hormonal crosstalk** between auxin, ethylene and cytokinin via the action of the *PLS* gene was analyzed using **kinetic modeling** (Liu et al., 2010a). On the basis of the model structure of the **hormonal crosstalk network**, relationships between auxin biosynthesis pathway(s) and PLS-regulated **hormonal crosstalk** can be analyzed. Although the molecular basis for auxin biosynthesis is poorly characterized, mathematical modeling reveals that the regulation of auxin biosynthesis by PLS peptide (PLSp) must be realized through its compounded effects with ethylene and cytokinin: PLSp cannot regulate auxin biosynthesis independently of the regulation of ethylene and cytokinin. This demonstrates that different structures of a **hormonal crosstalk network** may have different auxin concentration responses, revealing that a combined modeling and experimental analysis is a powerful tool for dissecting the causal relationship for the interactions between genes and **hormonal crosstalk** (Liu et al., 2010a). In addition, the **hormonal crosstalk network** constructed by iteratively combining experimental data and mathematical modeling (Liu et al., 2010a) can further integrate novel experimental data (Liu et al., 2013). Thus, through the cycle of modeling predictions and novel experimental measurements, the **hormonal crosstalk network** can be used to analyse the functions of hormone signals in root development and predict new experiments.

The role of the crosstalk between auxin and cytokinin signaling in specifying the root architecture of Arabidopsis has also been modeled using **kinetic modeling** (Muraro et al., 2011, 2013). It is found that tissue-specific oscillations in gene expression can be understood based on the joint activity of auxin and cytokinin (Muraro et al., 2013). These results reveal that **hormonal crosstalk** can be a mechanism explaining time-dependent dynamics such as oscillations, although other mechanisms may also generate oscillations in gene expression (Bujdoso and Davis, 2013; Rue and Garcia-Ojalvo, 2013).

The crosstalk between auxin and BRs signaling has also been analyzed using a Boolean logic approach (Sankar et al., 2011). An advantage of this approach is that it does not require kinetic parameters, which are usually not available (Liu et al., 2010b). However, the underlying assumptions of Boolean logic and their relevance to biological reality should be carefully assessed when applied to the modeling of **hormonal crosstalk networks**.

## Perspectives

This review has focused on recent progress in (a) experimental measurements relating to hormonal interactions in the Arabidopsis root; (b) construction of **hormonal crosstalk networks** based on experimental data; and (c) combination of experimental and mathematical modeling for elucidating and predicting the roles of **hormonal crosstalk** in root development.

One of the important features for **hormonal crosstalk** in root development (Figure 2) is that change in one signaling component leads to change in other signaling components. Therefore, elucidating the regulation of one signaling component requires the development of novel modeling methodology (Liu et al., 2014).

**Figure 3:**
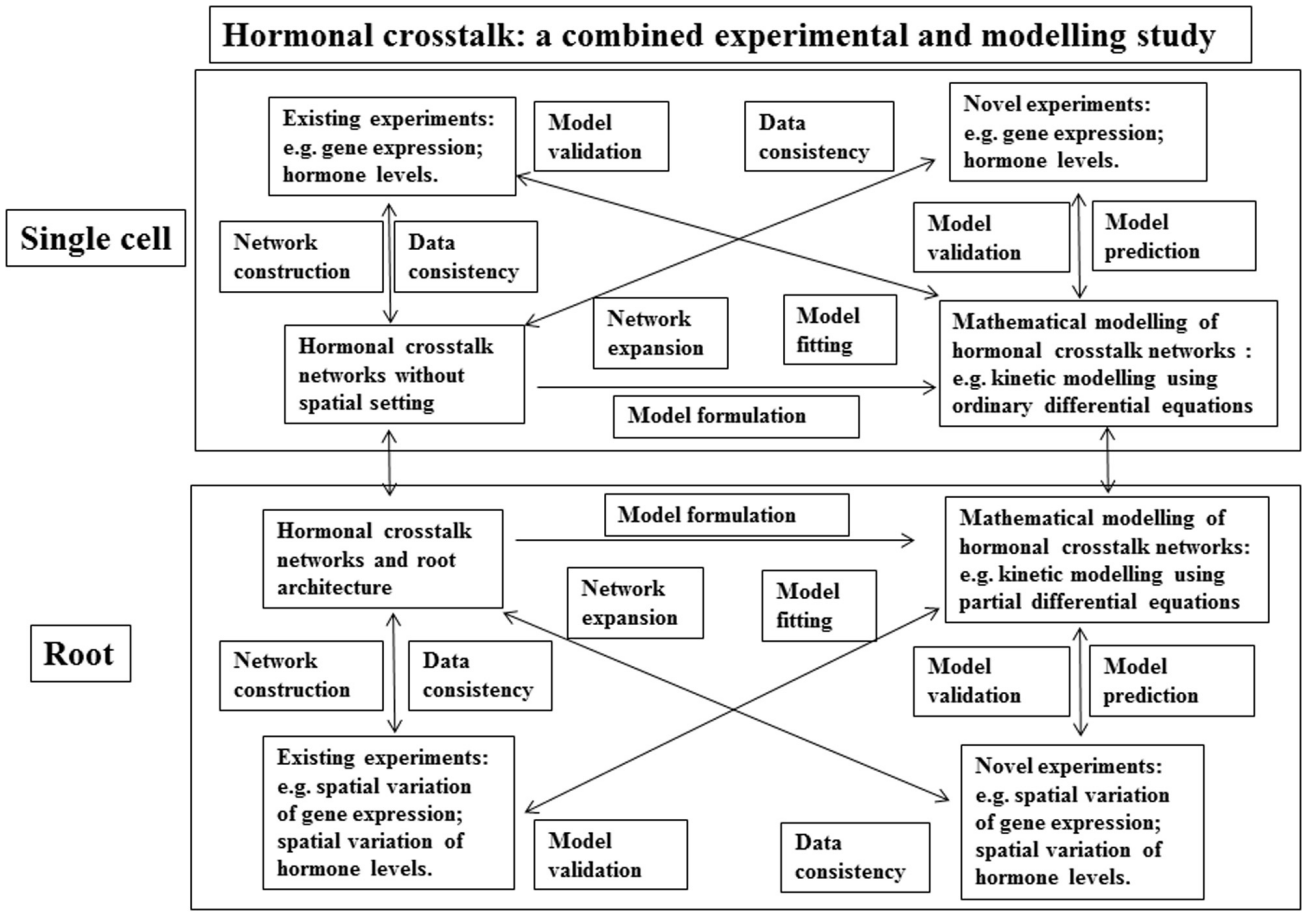
**A schematic description of a methodology for a combined experimental and modeling study on hormonal crosstalk in root development**. Upper panel: Hormonal crosstalk networks are constructed using existing experimental data. The networks are used to check the consistency of existing experimental data and they are also used to formulate mathematical models such as models described using ordinary differential equations. The mathematical models are validated using the existing experimental data. Then the mathematical models are used to predict novel experiments. The data acquired using the novel experiments are used to expand the hormonal crosstalk networks and to validate the mathematical models again. The expanded hormonal crosstalk networks are used to check the consistency of the novel experiments, and they are also used to develop novel mathematical models. Lower panel: the same as the upper panel, but root architecture is included.

An additional important aspect of hormonal signaling is its spatiotemporal dynamics. Understanding the roles of hormones in root development needs an analysis of the dynamics of **hormonal crosstalk networks** in spatial settings within a root. Here we propose a methodology that combines experimental data and mathematical modeling to study the spatiotemporal dynamics of hormonal signaling in root development (Figure 3). The study of **hormonal crosstalk** of auxin, ethylene and cytokinin in the non-spatial setting of a single cell has demonstrated that the methodology described in Figure 3 is a powerful tool (Liu et al., 2010a, 2013).

For the spatiotemporal modeling of **hormonal crosstalk** in the root, the important spatial aspects include (a) mechanisms of PIN polarity and auxin transport (van Berkel et al., 2013); (b) the relationship between auxin distribution and experimental observation of PIN polarity (Grieneisen et al., 2007); and (c) integration of multi-scale root systems (Hill et al., 2013). In addition to what we have discussed in the review, these aspects should be integrated when the root is modeled as an integrative system in space and time.

### Conflict of interest statement

The authors declare that the research was conducted in the absence of any commercial or financial relationships that could be construed as a potential conflict of interest.
